# Facilitators and barriers to adventurous play in British preschool-aged children: cross-sectional findings from the British Preschool Children’s Play Survey

**DOI:** 10.1136/bmjph-2025-004530

**Published:** 2026-09-04

**Authors:** Tom Rance, Brooke Oliver, Helen F Dodd, Kathryn R Hesketh

**Affiliations:** 1 IMS Epidemiology, University of Cambridge School of Clinical Medicine, Cambridge, UK; 2 School of Psychology and Clinical Language Sciences, University of Reading, Reading, UK; 3 Department of Public Health and Sport Sciences, University of Exeter, Exeter, UK; 4 UCL Institute of Child Health, London, UK

**Keywords:** Accidents, Public Health, Epidemiology

## Abstract

**Introduction:**

Adventurous play (AP), defined as play involving thrill, fear and uncertainty, has health and developmental benefits but can be challenging for parents to support. This study aimed to explore parent-reported facilitators and barriers to AP in preschool-aged children and whether these differ by sociodemographic characteristics.

**Methods:**

Cross-sectional data were from the British Preschool Children’s Play Survey, a nationally representative survey investigating play in British 2–4-year-olds. Parent-reported facilitators and barriers were analysed using a coding framework, with χ^2^ tests used to determine whether facilitators and barriers differed by sociodemographic characteristics (parent gender; child gender; ethnicity (white vs non-white); urbanicity (urban/suburban/rural); social grade (higher/lower); child age (2, 3 or 4 years); and child’s perceived motor skills).

**Results:**

Parents of 1166 children provided valid data. Safety (15.0%) and supervision (10.4%) were the most commonly reported facilitators, with child preferences (χ²=10.74, p=0.0008) and parental attitudes (χ²=5.60, p=0.006) more likely to be reported by white (vs minority ethnic) parents. Barriers were dominated by concerns about physical injury (18.8%) and safety (11.7%), with risk of injury reported by mothers more frequently than fathers (χ²=21.5, p<0.001) and parental attributes reported more frequently than fathers (χ²=17.2, p<0.001).

**Conclusions:**

Ensuring safety and supervision are important facilitators for children’s AP. Similar concerns about injury are perceived barriers. Given the benefits of AP for preschool-aged children, efforts to support AP opportunities should be prioritised. As the largest identified factor (both positive and negative) is children’s safety, future research efforts should acknowledge this.

**WHAT IS ALREADY KNOWN ON THIS TOPIC**

- Adventurous play (AP) offers important health and developmental benefits to children, yet opportunities for AP are declining. For preschool-aged children, where parental influence is particularly strong, evidence is lacking about what parents believe may encourage or prevent AP.

**WHAT THIS STUDY ADDS**

- This large, nationally representative study of parents identifies facilitators and barriers of preschool children’s AP and explores how these vary across sociodemographic groups. Results show that safety, adult supervision and concerns about physical injury are dominant themes for parents in relation to preschoolers’ AP.

**HOW THIS STUDY MIGHT AFFECT RESEARCH, PRACTICE OR POLICY**

- A deeper understanding of the parental facilitators and barriers that may influence AP in preschool-aged children will support the design of interventions and environments that offer developmentally rich AP opportunities. For example, because physical injury was the most common barrier reported, it may be useful to investigate more thoroughly the association between AP and injury. Following this, interventions for parents could be created to help them assess and understand the risks associated with AP and the consequences of restricting AP in young children.

## Introduction

 Play is a universal childhood experience and a fundamental right of the child.^1^ Although many forms of play exist, adventurous play (AP), defined as play involving thrill, fear and uncertainty with a risk of physical injury,^2^ is associated with significant positive behavioural and health outcomes for children. For example, a study of Norwegian children aged 3–5 showed that AP was positively correlated with increased levels of physical activity (PA) and enjoyment,^3^ and AP tends to be associated with higher intensity outdoor PA in school aged children.^4,5^ Early childhood is a critical period for developing the fundamental gross motor skills that underpin later PA.^6^ AP offers the graded physical challenges through which children practise these skills, testing balance, coordination and strength at the limits of their current ability.^7^ Risk taking also allows children to test their physical and psychological limits, develop their individual tolerance thresholds and create boundaries to prevent injury throughout childhood.^8^ Moreover, engaging in greater AP has also been associated with positive affect and fewer symptoms of anxiety in British preschool-aged and school-aged children.^9–11^

Despite these benefits, opportunities for AP are declining.^12–14^ The British Children’s Play Survey (BCPS) showed that 5–11-year-old children in Britain go out in their neighbourhood without parental supervision 2 years later than their parents’ generation did.^15^ Moreover, children in the UK now spend approximately 50% less time outside than a generation ago, resulting in fewer opportunities for outdoor AP.^16^ In line with this, data from both school-aged^15^ and preschool-aged^17^ children show that the most common place for children to play is at home, yet the home environment is also consistently rated as the place their children play least adventurously by parents. There are also inequalities in play opportunities: in children aged 2–4 years, girls and those from ethnic minority groups spend less time playing compared with boys and white children, respectively,^17^ with similar findings seen in research conducted with preschoolers in the USA.^18^ Evidence also suggests that preschool-aged children in urban areas spend less time playing than those in rural areas, suggesting geographic differences emerge from an early age.^17^ In contrast, evidence from the UK’s Millennium Cohort Study indicated that 7-year-old children who were from lower-income families and those who were living in close proximity to both family friends and family were more likely to have greater independent outdoor play.^19^

To improve children’s opportunities for AP, it is important to understand both non-modifiable and modifiable factors that influence access to AP. A previously conducted qualitative systematic review found that commonly reported barriers to AP were parental concerns regarding health and safety,^20^ mirrored in British school-aged children, where four of the top five barriers perceived by parents to inhibit children engaging in AP were concerns regarding safety (ie, safety of society, risk of injury, general safety concerns and safety of roads).^21^ Similar findings have been identified in schools with teachers,^22^ with a study of Australian and Norwegian teachers finding that AP was restricted within schools for fear of children’s safety and the potential for a playground injury.^23^

While there is an emerging understanding of the barriers and facilitators of AP for school-aged children, little is known about what parents perceive as factors influencing AP in preschool-aged children (ie, under 5s). This is particularly important as parents are key gatekeepers for preschoolers’ behaviours. Thus, this study aimed to explore parent-reported facilitators and barriers to AP in a sample of British 2–4-year-olds. Given that a range of demographic and geographic factors have been shown to be associated with time spent in AP,^19^ a secondary aim was to explore whether facilitators and barriers differed by sociodemographic factors.

## Methods

### Participants

Data were from the British Preschool Children’s Play Survey (BPCPS), a cross-sectional nationally representative online survey conducted to investigate play behaviours in UK preschool-aged children (2–4 years old). The BPCPS is closely aligned with the BCPS which focused on children aged 5–11 years^15^ to allow comparison across datasets. Detailed explanations of measures undertaken in the BCPS and BPCPS and descriptive statistics from the surveys are provided elsewhere.^15,17^ The full survey is provided in the online supplemental material.

### Procedure

Data were collected by YouGov through their online panel, which includes over 1 million adults living in the UK. YouGov recruit using active sampling to ensure diversity, inviting members of their research panel to complete online surveys. Approximately 14 275 parents were pre-identified as having a child aged 2–4 and eligible to participate and subsequently were sent the invitation email. Those who expressed interest and consented were provided with the survey via email. The survey included questionnaires about the mental health of children and parents, measures of parents’ attitudes to risk, children’s play (time), including AP, childcare attendance, sociodemographic and geographic information. Participants were rewarded with YouGov points, which could be exchanged for payment. When our a priori sample size was reached (n~1100), sampling ceased and the study closed, meaning no further participants were able to consent. For the present study, the participating sample were recruited to be approximately representative of the national population and then weighted based on age, gender, social class, region, and level of education to the national profile of adults living in the UK.

### Variables of interest

To assess facilitators and barriers of AP, parents provided free text answers in response to the following questions:

1. “Which factors, if any, *allow you* to let/encourage your child to play in an adventurous way?”
2. “Which factors, if any, *make it difficult* for you to let/encourage your child to spend more time playing in an adventurous way?”

Detailed sociodemographic information was used to generate variables to assess whether responses differ across sociodemographic factors. These were chosen to reflect factors deemed to be potentially important for children’s AP: parent gender (M/F); child gender (M/F); urbanicity (urban/suburban/rural); social grade (higher/lower) and child age (2, 3 or 4 years). For ethnic group, we categorised children as belonging to either white or minority ethnic group, as we were unable to analyse distinct minority ethnic groups due to very low numbers of children in each group. Additionally, we assessed a child’s perceived motor skills in comparison to their peers using the previously validated question, “How would you rate their physical development (eg, motor skills) compared with other children of the same age?”. Responses were collected on a three-point Likert scale ranging from less able than their peers to more able than their peers with similarly able in the middle. This was included as children whose parents perceive them as less physically capable may be given fewer opportunities to play/adventurously.^24^

### Coding scheme

The framework for coding barriers and facilitators to AP was developed by Oliver *et al* using data from the BCPS.^25^ It aimed to systematically categorise parent responses into categories of barriers and facilitators of AP.

As parents were asked to provide both facilitators and barriers, the same factor could appear in both categories, since the absence of a barrier was often described as a facilitator and vice versa. Facilitators and barriers were coded separately for ease of interpretation, but some categories appear under both because they were viewed both positively and negatively by parents. For example, safety was reported as a facilitator when parents felt able to assure it, and as a barrier when they could not. Parental attitudes and beliefs referred to a parent’s expressed beliefs about the value of AP: most often its benefits for the child’s learning and development. Responses expressing a negative view, for example, that AP is unnecessary or irresponsible, were coded to the same category. Parent attributes referred to characteristics of the parent that shaped their encouragement of AP. These comprised self-described traits, such as being anxious. Parenting styles described the parents' own described behaviours or actions, that is, permissive or restrictive behaviours that actively influenced the ability of the child to engage in AP.

For each participant, free-text responses to the facilitator and barrier questions were coded against the framework. Where a parent gave more than one response, codes were assigned to all relevant categories. For example, an answer of ‘safety and weather’ was coded as present (1) for both safety and weather. All categories not mentioned in a response were coded as absent (0). Responses were coded by meaning rather than by the question under which they were written, so that a clear barrier written in answer to the facilitator question was coded as a barrier. Non-sensical/blank responses were assigned a code which were not analysed. This was separate to the coding for ‘no facilitators/barriers’.

### Procedure and reliability checks

The extent to which the BCPS coding framework was appropriate for the current dataset was assessed by coding a subset of the responses without reference to the BCPS coding framework. A de novo coding system was developed and compared with the existing framework categories. As the codes aligned well, the existing framework was deemed appropriate and subsequently used to code all responses; no responses fell outside of this coding framework.

Two coders (TR, BO) coded the dataset. To ensure the reliability and consistency of the coding process, a subset comprising ~20% of the dataset was randomly selected for coding by both coders. BO developed the original framework and provided training to the primary coder TR. Following the coding of this 20%, inter-rater reliability was evaluated to assess agreement between the coders. Mean percentage agreement was 99% (range 93–100%) and mean Cohen’s kappa across categories was 0.84 range (0.50–1). All kappa values less than 0.7 were reviewed by the coders (n=10).^26^ These low values only occurred in categories that had very little data, making the kappa unreliable.^18^ Any coding discrepancies were resolved through discussion until consensus was reached. Data from the primary coder were used for the main analyses.

### Data analysis

Data analysis was conducted using R and STATA. Descriptive statistics were generated to summarise characteristics of included parents and children. Summary statistics (total number and percentage of responses) were generated for each of the facilitators and barriers by category. χ^2^ tests for independence were then used to assess the relationship between the identified facilitators and barriers and each of the seven sociodemographic variables. A Bonferroni corrected alpha (0.05/7=0.007) was used to adjust for multiple comparisons.

## Results

Of the 1341 who started to complete the survey, 1166 parents and caregivers of children aged 2–4 years (mean child age=3.1 years, SD=0.8 years; 48% female) completed all measures and comprise the study sample. The demographic characteristics of the sample are shown in table 1 and roughly align with the latest UK census data.^27^ The majority of participants were parents or step-parents (96%) and are henceforth referred to as parents.

**Table 1:** Demographic characteristics of the sample

Characteristic	N	%
Respondent sex		
Male	398	34
Female	768	66
Child sex		
Male	604	52
Female	562	48
Respondent age (years)		
18–24	57	5
25–34	356	31
35–44	702	60
45–54	40	3
54+	11	1
Child age (years)		
2	327	28
3	418	36
4	421	36
Relationship to child		
Mother	738	63
Father	379	33
Stepparent	26	2
Grandparent	15	1
Child birth order		
First-born	632	54
Second-born	379	33
Third or more	155	13
Respondent ethnicity		
White	1003	88
Minority	141	12
Prefer not to say	22	2
Employment status		
Working full-time	592	51
Working part-time	311	27
Student	19	2
Retired	8	1
Unemployed or not working	181	16
Other	55	5
Education level		
Low	176	15
Medium	340	30
High	621	55
Marital status		
Married or living as married	980	84
Separated, divorced or widowed	51	4
Never married	135	12
Great Britain region		
England	1010	87
London	273	23
North	203	17
Midlands	112	10
East	137	12
South	285	24
Wales	54	5
Scotland	102	9
Location		
Urban	919	79
Town or fringe	128	11
Rural	113	10
Social class		
Middle class (ABC1)	739	63
Working class (C2DE)	427	37
Respondent disability		
Yes, limited a lot	131	11
Yes, limited a little	73	6
No	936	82
Child learning disability		
Yes	72	6
No	1000	86
Prefer not to say	38	3
Don’t know	56	5
Child physical disability		
Yes	26	2
No	1078	92
Prefer not to say	34	3
Don’t know	28	2

### Facilitators to AP

The top 10 facilitators for AP are shown in table 2. Safety was the most frequently reported facilitator (15.0%), with supervision (10.4%); the availability of play equipment (10.4%); parents’ parenting styles (10.2%) (supportive and permissive approaches); and parental attributes (10.1%) comprising the top five reported facilitators.

**Table 2:** Top 10 facilitators of adventurous play

	Facilitator	Percentage of parents who report as a facilitator
1	Safety	15.0
2	Supervision	10.4
3	Play equipment	10.4
4	Parenting styles	10.2
5	Parenting attributes	10.1
6	Attitudes and beliefs	8.3
7	Child preference	6.3
8	Risk of physical injury	6.0
9	Child attributes	4.4
10	Support of other children	4.2

χ² analyses indicated that parents of white ethnic backgrounds were more likely to report that child preferences (χ²=10.74, p=0.0008) and parental attitudes and beliefs were facilitators to AP (χ²= 5.60, p=0.006) compared with parents from minority ethnic backgrounds. Facilitators did not differ significantly by any other sociodemographic factors.

### Barriers to AP

The top 10 barriers for AP are shown in table 3. Risk of physical injury was the most commonly reported barrier, with 18.8% of parents citing it as a concern. Closely linked to this, general safety concerns were cited by 11.7% of parents.

**Table 3:** Top 10 barriers to adventurous play

	Barrier	Percentage of parents who report as a barrier
1	Risk of physical injury	18.8
2	Safety	11.7
3	Child attributes	7.5
4	Adventurous play areas	7.0
5	Supervision	6.3
6	Time	5.5
7	Accessibility	5.1
8	Parent attributes	4.3
9	Weather	4.2
10	Roads	3.9

Significant sociodemographic differences were identified for several barriers (table 4). Risk of physical injury (χ² = 21.5, p=<0.001) and parental attributes (χ²=17.2, p=<0.001) were more likely to be cited as barriers by female parents compared with males. Child attributes were more likely to be cited as a barrier to AP by parents who rated their child as being less physically able than their peers (vs being more or similarly able as peers) (χ² = 19.9, p=0.0002).

**Table 4:** Sociodemographic differences across facilitators and barriers (where differences identified)

		Facilitators		Barriers			
		Child preference (%)	Parent attitudes and beliefs (%)	Risk of injury (%)	Parent attributes (%)	None (%)	Child attributes (%)
Ethnicity	White	8	4				
	Minority ethnic	2	1				
Parent gender	Male			12	2	17	
	Female			22	6	10	
Motor skills	More able						6
	Similarly able						6
	Less able						18

The percentage of responses which indicated that there were no barriers to AP was 12.0% making it the second most reported response. Male parents were more likely to cite no barriers to AP (χ² = 12.9, p=0.001).

## Discussion

This is the first study to comprehensively assess facilitators and barriers to AP in preschool-aged children. Results from a large nationally representative survey conducted in Britain show that safety and adequate supervision are the most commonly reported facilitators, whereas concerns regarding injuries are the most frequently reported barrier. Facilitators and barriers were relatively consistent across sociodemographic groups, although differences by ethnicity, parental gender and motor skills were identified. Parents’ perceptions about which factors may help or hinder AP in preschool-aged children are largely consistent regardless of children’s backgrounds. Collectively, this provides an understanding of barriers and factors that facilitate AP, which can be used to tailor support and interventions to encourage greater AP in children under five.

The facilitators identified in this study, including safety, supervision, environmental provisions and parental facilitation, highlight the broad combination of factors that parents believe shape children’s opportunities for AP. As noted, parents were able to freely choose which factors they deemed to both facilitate *and* hinder their child’s AP. While it was not possible to capture exactly what each parent meant by safety and supervision, the finding that parental concerns around safety and supervision are perceived to be an important facilitator for children’s AP closely aligns with previous research.^20,21,28^ Indeed, parents suggested that knowing their preschool-aged child is safe and supervised facilitated their AP, likely because they feel more comfortable letting their child take risks within sight. This is in contrast to previous work in older children which suggests that parents’ restrictive supervision practices, driven primarily by safety fears regarding environmental dangers and inadequate visibility or monitoring, directly impacted the extent and type of children’s outdoor active play.^29^ Moreover, parental anxieties related to unsupervised outdoor play and the implementation of restrictive rules appeared to substantially constrain preschool children’s AP opportunities in an Australian study of 3–5-year olds.^30^ Indeed, in the UK BPCPS, children whose parents were more open to risk taking were likely to engage in greater AP.^31^ Further work is therefore required to better understand how parents of young children make judgements about what is and isn’t ‘safe’, and the extent to which these judgements then inhibit or enhance young children’s opportunities for AP.

Perhaps unsurprisingly, concerns around child safety and the risk of injury were the most reported barriers to AP, with over 30% of parents referring to one or other of these factors in their responses. Approximately one in five respondents in this study identified risk of physical injury as a key barrier to AP, which aligns with previous research in older children.^21,32–34^ Responses were coded as ‘Risk of physical injury’ when specified that the barrier related to the play activity itself and the prospect of the child being hurt while doing it. Where parents identified a specific concern, such as busy roads, this was captured by the relevant code.^25^ Responses coded as ‘Safety’ were those in which parents referred to safety in general terms, without specifying whether they meant the play, social or physical environment. Although unspecified, these responses likely reflect a composite of concerns spanning the physical environment, the people within it and the child’s own capabilities.

These concerns are reflected at a societal level, where a sustained focus on safety has reshaped the spaces in which children play. For example, changes to playground layout have become increasingly designed to minimise physical risk and prioritise safety over developmental needs. And yet, playground equipment that is designed to prevent injury often fails to provide the height or speed that children crave during thrilling AP, which can in fact lead to boredom and even riskier behaviour.^35^ This paradox, where excessive safety measures lead to unintended dangerous behaviours, is a recurring theme in playground safety research.^36,37^ It is also apparent in the present findings, with parents reporting inadequacy of AP areas as a barrier to play. A focus on safety may therefore deprive children of the opportunity to engage in the kind of challenging AP that helps them build essential skills. Moreover, very little research has been undertaken to investigate the objective risk of children sustaining an injury when engaging in AP, with current injury data insufficiently detailed to address this concern.^33^ Future research should focus on combining novel, detailed, specific clinical data with contextual information from families to explore the association between AP and injury. In addition, although in school-aged British children, four out of the top five barriers identified related to safety including concerns about societal safety, parents of preschool-aged children did not express the same concerns (ie, perceived stranger danger, road safety).^21^ This likely reflects the lower level of independent mobility younger children have.^38–41^ Research is required to understand how and why these parental safety concerns develop and manifest. With overall safety and injury prevention being key issues reported by parents of preschool and school-aged children, addressing these is an important step to developing ways to encourage greater AP in all children.

Parents who perceived their child to be less physically able than their peers were significantly more likely to identify their child’s attributes as a barrier to AP compared with those who perceived their child as similarly or more physically able (18% vs 6%). This is consistent with findings from school-aged children, where parents who had a child with a disability were more likely to identify child attributes as a barrier to AP than those whose child did not have a disability (37% vs 10%).^21^ There is some evidence to suggest that young children with impaired motor skills are more likely to suffer injuries requiring hospital visits than those with good motor skills.^42^ Exploring differences among children who are and are not living with disabilities and their parents is therefore warranted, and although beyond the scope of this work, may provide important evidence to support all children to engage in AP that is appropriate to their (developmental) ability.

Parental gender influenced the reporting of barriers to AP, particularly concerning the risk of physical injury and parental attributes, with mothers generally exhibiting greater caution compared with fathers; this is in-keeping with one study of 88 parent-preschool child dyads in the UK.^43^ Furthermore, while no gender effect was found for parents of school-aged children in overall risk tolerance, notable gender-specific variations for particular high-risk activities were found, with male caregivers demonstrating higher tolerance for ‘riskier’ activities.^43^ There were no differences in this cohort between the facilitators and barriers for male versus female children. This is in-keeping with one study of preschool children in the UK, which found no significant difference between parental risk tolerance of AP between male and female children.^44^

Finally, white parents were more likely to cite parental attitudes and beliefs and child preference as key facilitators to AP compared with parents from minority ethnic groups. The factors influencing play in preschool children from minority ethnic backgrounds remain less well understood, and should be a key area of future study. Research conducted with diverse groups is required to explore specific factors that shape experiences, including cultural and geographic differences. For example, interviews with parents of 0–3-year-olds from multi-ethnic backgrounds in the north of England cited barriers to outdoor play, including structural, community and individual factors: lack of knowledge about where to go, or how to get there and confidence in managing young children while outdoors, were coupled with fear of crime, antisocial behaviour and accidents.^45^ That work also showed how social and community influences could both positively (through positive social interactions, and practical support by others) and negatively (antisocial or inappropriate behaviours experienced in greenspace) impact children’s play,^45^ mirroring the concerns identified here. Barriers related to the built environment were prominent, including unsafe playgrounds, no garden or safe play space and poor accessibility. Fears about safety persisted even where greenspace was of high quality.^45^ Improving provision alone, therefore, is unlikely to increase use. Given preschool-aged children from minority ethnic backgrounds have been found to engage in less AP in comparison to white children,^9^ understanding the cultural and contextual factors that may prevent or encourage AP is vital to ensure health inequalities are not perpetuated.

### Strengths and limitations

Data were from a large, nationally representative sample of preschool-aged children’s parents, using active sampling at a population-level to minimise the effects of sampling bias. It includes data from a breadth of geographical locations, socioeconomic strata and ethnic groups. The use of an established coding framework^25^ enhances validity and allows comparison to other similar research. Reliability checks with dual independent coding were employed, and a high level of inter-rater reliability was found. However, the use of parent-report measures may be susceptible to social desirability bias, with parents providing responses they perceive as socially acceptable rather than their true beliefs. This may impact the accuracy and reliability of the reported factors, though the commonalities across this and other similar surveys suggest a broad range of views were captured. Combining all minority ethnic groups into one category likely risks missing key differences within the sample, but was done because of insufficient power within distinct groups, and still highlights the potential differences in experiences for white and minority ethnic families. Specific research to explore barriers and facilitators across different minority ethnic demographic groups is now required.

## Conclusion

For UK preschool-aged children, parental concerns regarding safety and the risk of injury are perceived to be key barriers to AP, whereas reported facilitators mirror these concerns by emphasising the importance of adequate safety and supervision. Given the benefits of AP for preschool-aged children, efforts to support AP opportunities should be prioritised. As the largest identified factor (both positive and negative) is children’s safety, research efforts should acknowledge this and seek to put mechanisms in place to explore the different facets of this factor. This might include implementing nationwide injury surveillance to understand the risk AP may present to young children,^46^ and how communities can make their play spaces more visible and welcoming to a wider range of families. In doing so, interventions may then be developed to safely encourage parents to provide appropriate opportunities for AP in preschool-aged children, supporting their health and well-being.

## Supplementary material

10.1136/bmjph-2025-004530 online supplemental appendix 1

## Data Availability

Data are available upon reasonable request.

## References

[1] United Nations UN convention on the rights of the child. https://www.unicef.org.uk/wp-content/uploads/2016/08/unicef-convention-rights-child-uncrc.pdf.

[2] Full article: categorising risky play—how can we identify risk‐taking in children’s play?. https://www.tandfonline.com/doi/full/10.1080/13502930701321733.

[3] Sando OJ, Kleppe R, Sandseter EBH (2021). Risky Play and Children’s Well-Being, Involvement and Physical Activity. Child Ind Res.

[4] Burdette HL, Whitaker RC, Daniels SR (2004). Parental report of outdoor playtime as a measure of physical activity in preschool-aged children. Arch Pediatr Adolesc Med.

[5] Brussoni M, Gibbons R, Gray C (2015). What is the Relationship between Risky Outdoor Play and Health in Children? A Systematic Review. Int J Environ Res Public Health.

[6] Bremer E, Cairney J (2018). Fundamental Movement Skills and Health-Related Outcomes: A Narrative Review of Longitudinal and Intervention Studies Targeting Typically Developing Children. Am J Lifestyle Med.

[7] Hansen Sandseter EB, Kleppe R, Ottesen Kennair LE (2023). Risky play in children’s emotion regulation, social functioning, and physical health: an evolutionary approach. Int J Play.

[8] Brussoni M, Olsen LL, Pike I (2012). Risky play and children’s safety: balancing priorities for optimal child development. Int J Environ Res Public Health.

[9] Hesketh KR, Dodd HF (2026). Are play and screen time associated with British preschoolers’ mental health? Cross-sectional findings from the British Preschool Children’s Play Survey. BMJ Open.

[10] Dodd HF, Nesbit RJ, FitzGibbon L (2023). Child’s Play: Examining the Association Between Time Spent Playing and Child Mental Health. Child Psychiatry Hum Dev.

[11] Dodd HF, Patterson E, Skripkauskaite S (2026). Longitudinal associations between play experiences and trajectories of preschoolers’ mental health from April-July, 2020. *JCPP Adv*.

[12] Singer DG, Singer JL, D’Agnostino H (2009). Children’s pastimes and play in sixteen nations: is free-play declining. Am J Play.

[13] Hofferth SL (2009). Changes in American children’s time - 1997 to 2003. Electron Int J Time Use Res.

[14] Shaw B, Bicket M, Elliott B Children’s independent mobility.

[15] Dodd HF, FitzGibbon L, Watson BE (2021). Children’s Play and Independent Mobility in 2020: Results from the British Children’s Play Survey. Int J Environ Res Public Health.

[16] Google Docs Everything to play for — raising the nation play commission.pdf. https://drive.google.com/file/d/1ckOdG4Rf7tdUUv4B2P6C7BoP5ei_ferT/view?usp=embed_facebook.

[17] Dodd HF, Hesketh K (2024). The British Preschool Children’s Play Survey: When, Where, and How Adventurously Do British Preschool-Aged Children Play?. J Phys Act Health.

[18] Dahl KL, Chen TJ, Nakayama JY (2024). Time Playing Outdoors Among Children Aged 3-5 Years: National Survey of Children’s Health, 2021. Am J Prev Med.

[19] Aggio D, Gardner B, Roberts J (2017). Correlates of children’s independent outdoor play: Cross-sectional analyses from the Millennium Cohort Study. Prev Med Rep.

[20] Nesbit RJ, Bagnall CL, Harvey K (2021). Perceived Barriers and Facilitators of Adventurous Play in Schools: A Qualitative Systematic Review. Children (Basel).

[21] Oliver BE, Nesbit RJ, McCloy R (2023). Adventurous play for a healthy childhood: Facilitators and barriers identified by parents in Britain. Soc Sci Med.

[22] Jerebine A, Fitton-Davies K, Lander N (2022). “Children are precious cargo; we don’t let them take any risks!”: Hearing from adults on safety and risk in children’s active play in schools: a systematic review. Int J Behav Nutr Phys Act.

[23] Little H, Sandseter EBH, Wyver S (2012). Early Childhood Teachers’ Beliefs about Children’s Risky Play in Australia and Norway. Contemp Issues Early Child.

[24] (2020). Stone M, Cawley J, Houser N, Kirk SF. Are Parental Perceptions of Risk and Attitudes Toward RiskTaking During Play Associated with Preschoolers’ Physical Activity and Physical Literacy? Vol. 23 No. 2 (2020): Outdoor Play and Early Learning. Vol. 23 No. 2 (2020): Outdoor Play and Early Learning..

[25] Oliver BE, Nesbit RJ, McCloy R (2022). Parent perceived barriers and facilitators of children’s adventurous play in Britain: a framework analysis. BMC Public Health.

[26] Brennan P, Silman A (1992). Statistical methods for assessing observer variability in clinical measures. BMJ.

[27] Office for National Statistics Census-based statistics UK: 2021. https://www.ons.gov.uk/peoplepopulationandcommunity/populationandmigration/populationestimates/datasets/censusbasedstatisticsuk2021.

[28] Waddington KC, Pearson ES (2022). Parental perspectives on the barriers and facilitators to risky-play in children. J Multidiscip Res Trent.

[29] Valentine G, McKendrck J (1997). Children’s outdoor play: exploring parental concerns about children’s safety and the changing nature of childhood. Geoforum.

[30] Wiseman N, Harris N, Downes M (2019). Preschool children’s preferences for sedentary activity relates to parent’s restrictive rules around active outdoor play. BMC Public Health.

[31] Tyzhuk K, Dodd HF, Hesketh KR (2025). Parental tolerance of risk and associations to adventurous play in British preschool-aged children. Prev Med Rep.

[32] Beaulieu E, Beno S (2024). Healthy childhood development through outdoor risky play: Navigating the balance with injury prevention. Paediatr Child Health.

[33] Rance T, Ramchandani P, Hesketh KR (2025). Risky play: our children need more. Arch Dis Child.

[34] Malone K (2007). The bubble‐wrap generation: children growing up in walled gardens. Environ Educ Res.

[35] Ball DJ (2004). Policy issues and risk-benefit trade-offs of “safer surfacing” for children’s playgrounds. Accid Anal Prev.

[36] Risk, challenge and safety: implications for play quality and playground design. https://www.researchgate.net/publication/241717477_Risk_challenge_and_safety_Implications_for_play_quality_and_playground_design.

[37] Herrington S, Nicholls J (2007). Outdoor play spaces in Canada: The safety dance of standards as policy. Crit Soc Policy.

[38] Loebach J, Sanches M, Jaffe J (2021). Paving the Way for Outdoor Play: Examining Socio-Environmental Barriers to Community-Based Outdoor Play. Int J Environ Res Public Health.

[39] Jerebine A, Mohebbi M, Lander N (2024). Playing it safe: The relationship between parent attitudes to risk and injury, and children’s adventurous play and physical activity. Psychol Sport Exerc.

[40] Clements R (2004). An Investigation of the Status of Outdoor Play. Contemp Issues Early Child.

[41] Carver A, Timperio A, Crawford D (2008). Playing it safe: the influence of neighbourhood safety on children’s physical activity. A review. Health Place.

[42] Myhre MC, Thoresen S, Grøgaard JB (2012). Familial factors and child characteristics as predictors of injuries in toddlers: a prospective cohort study. BMJ Open.

[43] Smith AD, Dodd HF, Ricardo L (2024). Gender Differences in Caregivers’ Attitudes to Risky Child Play in Britain: A Cross-Sectional Study. J Phys Act Health.

[44] Ryan ZJ, Stockill H, Nesbit RJ (2024). Parents’ Perception of Risk in Play: Associations with Parent and Child Gender. J Child Fam Stud.

[45] Cronin-de-Chavez A, Islam S, McEachan RRC (2019). Not a level playing field: A qualitative study exploring structural, community and individual determinants of greenspace use amongst low-income multi-ethnic families. Health Place.

[46] RCPCH (2026). State of child health 2026: injuries. https://www.rcpch.ac.uk/work-we-do/state-of-child-health/injuries.

