# Supplementary material for: Facilitators and barriers to adventurous play in British preschool-aged children: cross-sectional findings from the British Preschool Children’s Play Survey
Source: BMJ Public Health. 2026 Sep 4;4(3):e004530. doi: 10.1136/bmjph-2025-004530 (PMC13548242; doi:10.1136/bmjph-2025-004530)
Supplement: Supplementary file 1 [file bmjph-4-3-s001.pdf]

QUESTIONNAIRE

*Question type: **Pdl***
#Question display logic:
if pdl.Omnibus_Parents.last > months(1) and only asked
[Varlabel - Parents Omni One]

[Omnibus_Parents] Are you the parent/ guardian of children of any of the following ages? (Please select all that apply. If you do not have any children, please select the "Not applicable" option)

<1>	Under 6 months old	<12>	10 years old
<2>	Between 6 and 12 months old	<13>	11 years old
<3>	1 year old	<14>	12 years old
<4>	2 years old	<15>	13 years old
<5>	3 years old	<16>	14 years old
<6>	4 years old	<17>	15 years old
<7>	5 years old	<18>	16 years old
<8>	6 years old	<19>	17 years old
<9>	7 years old	<20>	18 years old
<10>	8 years old	<21>	Older than 18 years old
<11>	9 years old	<99 fixed xor>	Not applicable - I do not have any children

*Question type: **Single***
#Question display logic:
if 0

[Omnibus_Parents_recoded] Omnibus_Parents recoded - hidden variable

<1>	2
<2>	3
<3>	4

*Question type: **Text***

This survey is on the topic of your child's wellbeing, what they do in their spare time and your thoughts about children taking risks, the results will be used to inform our client.

Your YouGov Account will be credited with 50 points for completing the survey.

We have tested the survey and found that, on average it takes around XX minutes to complete. This time may vary depending on factors such as your Internet connection speed and the answers you give.

Please click the forward button below to continue.

YouGov plc is a public limited company incorporated in England and Wales with company registration number 0367311 and with its registered office at 50 Featherstone Street, London, EC1Y 8RT. Website: www.yougov.com Tel: +44 207 012 6000 VAT Registration Number: 991 2281 09

*Question type: **Text***

The button to move forward will appear after 15 seconds. Once it appears, please click this to continue.

*Question type: **Pdl***
#Question display logic:
if pdl.profile_marital_stat.last > months(1) and only asked
[Varlabel - Marital status]

[profile_marital_stat] What is your marital status?

<1> Married/ living with partner/ civil partnership
<2> Separated/ Divorced/ Widowed
<3> Never married

*Question type: **Pdl***
#Question display logic:
if 0
[Varlabel - Gender]

[profile_gender] Are you...?

<1> Male
<2> Female

*Question type: **Pdl***
#Question display logic:
if pdl.profile_education_level.last > months(1) and only asked
[Varlabel - Education qualification (highest attained)]

[profile_education_level] What is the highest educational or work-related qualification you have?

<1> No formal qualifications
<2> GCSE, Apprenticeship, City & Guilds certificate
<3> GCE A level or Higher Certificate/ Scottish Higher Certificate
<4> Professional diploma (e.g. nursing, teaching)
<5> University degree or graduate qualification

*Question type: **Pdl***
#Question display logic:
if pdl.profile_work_stat.last > months(1) and only asked
[Varlabel - Employment Status Main]

[profile_work_stat] Which of these applies to you?

<1> Employed full time (30 or more hours per week)

YouGov plc is a public limited company incorporated in England and Wales with company registration number 0367311 and with its registered office at 50 Featherstone Street, London, EC1Y 8RT. Website: www.yougov.com Tel: +44 207 012 6000 VAT Registration Number: 991 2281 09

<2>	Employed part time (8-29 hours a week)
<3>	Unemployed/ not working
<4>	Other (retired, full time student)

[profile_work_PA] Which of these applies to you?

We would like to know about the type and amount of physical activity involved in your work (Please choose one option that best corresponds to your occupation in the last year)

<1>	Sedentary – spend most of the time sitting (e.g. in an office)
<2>	Standing – spend most of the time standing or walking, but doesn't include intense physical effort (e.g. hairdresser, guard)
<3>	Physical work - includes physical effort including handling heavy objects/ use of tools (e.g. plumber, cleaner, nurse)
<4>	Heavy Manual work – includes vigorous activity, handling very heavy objects (e.g. construction worker)
<5>	Not in employment

[profile_parent_PA] In a typical week during the past 12 months, how many hours/ minutes did you spend on each of the following activities (put an 0 for none)

		Spring/Summer	Autumn/Winter
<1>	Walking, to work or for leisure		
<2>	Cycling, to work or for leisure etc.		
<3>	Other physical exercise (e.g. swimming, running, club sport)		

Base: All GB parents of children aged 2-4
Question type: ***Single***
[Varlabel - Number of children]

[Profile_fam_children] How many children do you have in total?

<1>	1
<2>	2
<3>	3
<4>	4 or more

For the following questions, please think about your child aged $Omnibus_Parents_recoded. If you have more than one child aged $Omnibus_Parents_recoded, please think about your oldest child aged $Omnibus_Parents_recoded.

YouGov plc is a public limited company incorporated in England and Wales with company registration number 0367311 and with its registered office at 50 Featherstone Street, London, EC1Y 8RT. Website: www.yougov.com Tel: +44 207 012 6000 VAT Registration Number: 991 2281 09

Base: All GB parents of children aged 2-4
*Question type: **Single***
[Varlabel - Relationship to child]

Which ONE of the following best describes your relationship with this child?

<1 if profile_gender ==2>	Mother
<2 if profile_gender ==1>	Father
<3 if profile_gender ==2>	Step-mother
<4 if profile_gender ==1>	Step-father
<6 if profile_gender ==2>	Grandmother
<7 if profile_gender ==1>	Grandfather
<955 fixed>	Other (open [GUC_Q2_open1]) [open] please specify

Base: All GB parents of children aged 2-4
*Question type: **Single***
#row order: randomize
[Varlabel - Primary caregiver]

Still thinking about your child aged $Omnibus_Parents_recoded.....

Which ONE of the following BEST describes who this child's primary care giver is?

<1>	I am this child's primary caregiver
<2>	Someone else is this child's primary caregiver
<3>	I share the role of being the child's primary caregiver with someone else

Base: All GB parents of children aged 2-4
*Question type: **Single***
#row order: randomize
[Varlabel - Child Gender]

Thinking about your oldest child aged $Omnibus_Parents_recoded....

What is the gender of this child?

<1>	Male
<2>	Female

What is your **child's** ethnicity?

<3>	Asian or Asian British
<2>	Black, Black British, Caribbean or African
<4>	Chinese or Chinese British
<1>	White
<5>	Mixed or multiple ethnicities
<6>	Other

YouGov plc is a public limited company incorporated in England and Wales with company registration number 0367311 and with its registered office at 50 Featherstone Street, London, EC1Y 8RT. Website: www.yougov.com Tel: +44 207 012 6000 VAT Registration Number: 991 2281 09

Base: All GB parents of children aged 2-4
Question type: ***Single***
[Varlabel - Birth order]

Thinking about your child aged $Omnibus_Parents_recoded...

What is the birth order of this child?

<1 if GUC_Q in [1,2,3,4,5,6,7]>	First born
<2 if GUC_Q in [2,3,4,5,6,7]>	Second born
<3 if GUC_Q in [3,4,5,6,7]>	Third born
<4 if GUC_Q in [4,5,6,7]>	Fourth born or later
<955 fixed>	Other (open [XX_open1]) [open] please specify

Base: All GB parents of children aged 2-4
Question type: ***Single***
[Varlabel - Learning difficulties, mental health problems or physical disabilities]

For the following question, we have provided you with a 'Prefer not to say' option if you would rather not share your experiences.

Still thinking about your child aged $Omnibus_Parents_recoded...
Does your child have any diagnosed with learning difficulties, neuro diversity or SEND?
(Please select the option that best applies. If the answer is yes, please provide brief details).

<1>	Yes (open [GUC_Q6_open1]) [open] please specify
<2>	No
<977 fixed xor>	Don’t know
<933 fixed xor>	Prefer not to say

Still thinking about your child aged $Omnibus_Parents_recoded...
Does your child have any physical disabilities?
(Please select the option that best applies. If the answer is yes, please provide brief details).

<1>	Yes (open [GUC_Q6_open1]) [open] please specify
<2>	No
<977 fixed xor>	Don’t know
<933 fixed xor>	Prefer not to say

Compared to other children of the same age, how would you rate your child’s physical development (e.g. motor skills):

<1>	Much less able than their peers
<2>	Slightly less able than their peers
<3>	About the same as their peers
<4>	Slightly more able than their peers
<5>	Much more able than their peers

YouGov plc is a public limited company incorporated in England and Wales with company registration number 0367311 and with its registered office at 50 Featherstone Street, London, EC1Y 8RT. Website: www.yougov.com Tel: +44 207 012 6000 VAT Registration Number: 991 2281 09

Does your child attend (choose all that apply)

		Number of hours/week	Number of days/week
<1>	Primary or infant school		
<2>	Preschool		
<3>	Nursery		
<4>	Informal care (e.g. grandparents)		
<5>	Other		

Does your child's childcare or school have an outdoor space to play?

<1> Yes, it does
<2> No, it doesn't
<977 fixed xor> Don’t know
<944 fixed xor> Not applicable - My child doesn't go to school

Does your child attend forest school?

<1> Yes, at school/ childcare
<2> Yes, at another setting
No
Don’t know

How do you usually make short trips with your child (by short trips we mean less than half a mile)?

<1> By car
<2> On public transport
<3> Walking with my child in a buggy
<4> My child walks with me
<5> Bicycling with my child in a seat
<6> My child cycles/ scoots themselves
<977 fixed xor> Other – please state

Base: All GB parents of children aged 2-4
*Question type: **Single***
[Varlabel - Frequency of organised activities a week]

Thinking about the last 6 months (i.e. since June 2022)... Outside of formal and informal childcare, approximately how many times per week did your child take part in any organised activities, aside of formal care (these activities are sometimes called “extra-curricular activities”, such music classes, mother-child swimming classes etc.)?

<1> Never
<2> Once a week or less often
<3> 2 to 3 times a week
<4> 4 to 5 times a week

YouGov plc is a public limited company incorporated in England and Wales with company registration number 0367311 and with its registered office at 50 Featherstone Street, London, EC1Y 8RT. Website: www.yougov.com Tel: +44 207 012 6000 VAT Registration Number: 991 2281 09

<5>	6 to 7 times a week
<6>	8 times a week or more
<977 fixed xor>	Don’t know/ can't recall

Base: All GB parents of children aged 2-4 whose child ever takes part in organised activity
*Question type: **Single***
#Question display logic:
if GUC_Q8 in [2,3,4,5,6]
[Varlabel - Frequency of organised activities involving energetic physical activity]

You previously said your child aged $Omnibus_Parents_recoded took part in organised activities approximately $X in the last 6 months.

Approximately how many times per week did your child <u>take part in energetic physical activity</u> while participating in organised activities (e.g. swimming class, gymnastics, rugby tots etc.)?

<1>	Never
<2 if GUC_ in [2,3,4,5,6]>	Once a week or less often
<3 if GUC_ in [3,4,5,6]>	2 to 3 times a week
<4 if GUC_ in [4,5,6]>	4 to 5 times a week
<5 if GUC_ in [5,6]>	6 to 7 times a week
<6>	8 times a week or more
<977 fixed xor>	Don’t know/ can't recall

#option display logic:
<6> - If [GUC_Q8] - 8 times a week or more is selected [if GUC_Q8 == 6]

*Question type: **Text***

The following questions will ask about where your child aged $Omnibus_Parents_recoded plays at different times of the year, how long they spend playing in these places, and how adventurously they play.

By 'play', we mean an activity that a child does for enjoyment when there is no serious or practical purpose. Formal lessons / classes do <u>not</u> count as play here, but playing with a ball with friends or parents <u>does</u> count as play. Please think about play in children's <u>day to day lives,</u> NOT when they are on holidays, or in school, childcare or organised sports, or when they are using a screen (e.g. watching TV, playing on a games on tablets etc.).

The button to move forward will appear after 20 seconds. Once it appears, please click this to continue.

Base: All GB parents of children aged 2-4
*Question type: **Grid***
#max number of choices per page: 4
[Varlabel - Frequency of playing Autumn and Winter]

[GUC_Q12] Thinking about the places that your child typically plays in <u>Autumn and Winter</u> (i.e. from September to February)...

YouGov plc is a public limited company incorporated in England and Wales with company registration number 0367311 and with its registered office at 50 Featherstone Street, London, EC1Y 8RT. Website: www.yougov.com Tel: +44 207 012 6000 VAT Registration Number: 991 2281 09

How frequently, if at all, does your child play in each of the following places in Autumn and Winter? (Please select one option on each row)

-[GUC_Q12_1] Varlabel:At home or in other people's homes	At home or in other people's homes
-[GUC_Q12_2] Varlabel:Outside at home or at other people's homes (e.g. in a garden, yard, a balcony etc.)	Outside at home or at other people's homes (e.g. in a garden, yard, a balcony etc.)
-[GUC_Q12_3] Varlabel:At a playground	At a playground
-[GUC_Q12_4] Varlabel:In trees/ woodlands/ grassy spaces (not including a garden at home or in other people's homes)	In trees/ woodlands/ grassy spaces (not including a garden at home or in other people's homes)
-[GUC_Q12_5] Varlabel:In the street or public space close to your home (e.g. in your street, on the pavement in front of your home etc.) (not including local playgrounds)	In the street or public space close to your home (e.g. in your street, on the pavement in front of your home etc.) (not including local playgrounds)
-[GUC_Q12_6] Varlabel:Outdoors near water (e.g.at a beach, in the sea, near a river, lake or cliffs etc.)	Outdoors near water (e.g.at a beach, in the sea, near a river, lake or cliffs etc.)
-[GUC_Q12_7] Varlabel:At indoor play centres and pools (e.g. soft play, trampoline parks, swimming pools etc.)	At indoor play centres and pools (e.g. soft play, trampoline parks, swimming pools etc.)
<1>	Everyday
<2>	4 to 6 times a week
<3>	2 to 3 times a week
<4>	Once a week
<5>	Once a month
<6>	Less than once a month

YouGov plc is a public limited company incorporated in England and Wales with company registration number 0367311 and with its registered office at 50 Featherstone Street, London, EC1Y 8RT. Website: www.yougov.com Tel: +44 207 012 6000 VAT Registration Number: 991 2281 09

<7>	Never
<977 fixed xor>	Don't know/ can't recall

Base: All GB parents of children aged 2-4 whose child ever plays in any of the listed places
*Question type: **Grid***
#max number of choices per page: 4
#Question display logic:
if GUC_Q12_1 in [1,2,3,4,5,6] or GUC_Q12_2 in [1,2,3,4,5,6] or GUC_Q12_3 in [1,2,3,4,5,6] or GUC_Q12_4 in [1,2,3,4,5,6] or GUC_Q12_5 in [1,2,3,4,5,6] or GUC_Q12_6 in [1,2,3,4,5,6] or GUC_Q12_7 in [1,2,3,4,5,6]
[Varlabel - Time spent playing in Autumn and Winter]

[GUC_Q13] You previously said that your child spends time playing in the following places in Autumn and Winter (i.e. from September to February)...

Thinking about a typical day that they play in each place, for approximately how long does your child play in each place in Autumn and Winter? (Please select one option on each row)

-[GUC_Q13_1 if GUC_Q12_1 in [1,2,3,4,5,6]] Varlabel:At home or in other people's homes	At home or in other people's homes
-[GUC_Q13_2 if GUC_Q12_2 in [1,2,3,4,5,6]] Varlabel:Outside at home or at other people's homes (e.g. in a garden, yard, a balcony etc.)	Outside at home or at other people's homes (e.g. in a garden, yard, a balcony etc.)
-[GUC_Q13_3 if GUC_Q12_3 in [1,2,3,4,5,6]] Varlabel:At a playground	At a playground
-[GUC_Q13_4 if GUC_Q12_4 in [1,2,3,4,5,6]] Varlabel:In trees/ woodlands/ grassy spaces (not including a garden at home or in other people's homes)	In trees/ woodlands/ grassy spaces (not including a garden at home or in other people's homes)
-[GUC_Q13_5 if GUC_Q12_5 in [1,2,3,4,5,6]] Varlabel:In the street or public space close to your home (e.g. in your street, on the pavement in front of your home etc.) (not including local playgrounds)	In the street or public space close to your home (e.g. in your street, on the pavement in front of your home etc.) (not including local playgrounds)
-[GUC_Q13_6 if GUC_Q12_6 in [1,2,3,4,5,6]] Varlabel:Outdoors near water (e.g.at a beach, in the sea, near a river, lake or cliffs etc.)	Outdoors near water (e.g.at a beach, in the sea, near a river, lake or cliffs etc.)
-[GUC_Q13_7 if GUC_Q12_7 in [1,2,3,4,5,6]] Varlabel:At indoor play centres and pools (e.g. soft	At indoor play centres and pools (e.g. soft play, trampoline parks, swimming pools etc.)

YouGov plc is a public limited company incorporated in England and Wales with company registration number 0367311 and with its registered office at 50 Featherstone Street, London, EC1Y 8RT. Website: www.yougov.com Tel: +44 207 012 6000 VAT Registration Number: 991 2281 09

play, trampoline parks, swimming pools etc.)	
<1>	Less than half an hour
<2>	Half an hour to 1 hour
<3>	1 to 2 hours
<4>	2 to 3 hours
<5>	3 to 4 hours
<6>	More than 4 hours
<977 fixed xor>	Don’t know/ can't recall

Base: All GB parents of children aged 2-4
*Question type: **Grid***
#max number of choices per page: 4
[Varlabel - Frequency of playing Spring and Summer]

[GUC_Q14] Thinking about the places that your child typically plays in <u>Spring and Summer</u> (i.e. from March to August)...

How frequently, if at all, does your child play in each of the following places in Spring and Summer? (Please select one option on each row)

-[GUC_Q14_1] Varlabel:At home or in other people's homes	At home or in other people's homes
-[GUC_Q14_2] Varlabel:Outside at home or at other people's homes (e.g. in a garden, yard, a balcony etc.)	Outside at home or at other people's homes (e.g. in a garden, yard, a balcony etc.)
-[GUC_Q14_3] Varlabel:At a playground	At a playground
-[GUC_Q14_4] Varlabel:In trees/ woodlands/ grassy spaces (not including a garden at home or in other people's homes)	In trees/ woodlands/ grassy spaces (not including a garden at home or in other people's homes)
-[GUC_Q14_5] Varlabel:In the street or public space close to your home (e.g. in your street, on the pavement in front of your home etc.) (not including local playgrounds)	In the street or public space close to your home (e.g. in your street, on the pavement in front of your home etc.) (not including local playgrounds)

YouGov plc is a public limited company incorporated in England and Wales with company registration number 0367311 and with its registered office at 50 Featherstone Street, London, EC1Y 8RT. Website: www.yougov.com Tel: +44 207 012 6000 VAT Registration Number: 991 2281 09

-[GUC_Q14_6] Varlabel:Outdoors near water (e.g.at a beach, in the sea, near a river, lake or cliffs etc.)	Outdoors near water (e.g.at a beach, in the sea, near a river, lake or cliffs etc.)
-[GUC_Q14_7] Varlabel:At indoor play centres and pools (e.g. soft play, trampoline parks, swimming pools etc.)	At indoor play centres and pools (e.g. soft play, trampoline parks, swimming pools etc.)
<1>	Everyday
<2>	4 to 6 times a week
<3>	2 to 3 times a week
<4>	Once a week
<5>	Once a month
<6>	Less than once a month
<7>	Never
<977 fixed xor>	Don't know/ can't recall

Base: All GB parents of children aged 2-4 whose child ever plays in any of the listed places
*Question type: **Grid***
#max number of choices per page: 4
#Question display logic:
if GUC_Q14_1 in [1,2,3,4,5,6] or GUC_Q14_2 in [1,2,3,4,5,6] or GUC_Q14_3 in [1,2,3,4,5,6] or GUC_Q14_4 in [1,2,3,4,5,6] or GUC_Q14_5 in [1,2,3,4,5,6] or GUC_Q14_6 in [1,2,3,4,5,6] or GUC_Q14_7 in [1,2,3,4,5,6]
[Varlabel - Time spent playing in Spring and summer]

[GUC_Q15] You previously said that your child spends time playing in the following places in Spring and summer (i.e. from March to August)...

Thinking about a typical day that they play in each place, for approximately how long does your child play in each place in Spring and Summer? (Please select one option on each row)

-[GUC_Q15_1 if GUC_Q14_1 in [1,2,3,4,5,6]] Varlabel:At home or in other people's homes	At home or in other people's homes
-[GUC_Q15_2 if GUC_Q14_2 in [1,2,3,4,5,6]] Varlabel:Outside at home or at other people's homes (e.g. in a garden, yard, a balcony etc.)	Outside at home or at other people's homes (e.g. in a garden, yard, a balcony etc.)
-[GUC_Q15_3 if GUC_Q14_3 in [1,2,3,4,5,6]] Varlabel:At a playground	At a playground
-[GUC_Q15_4 if GUC_Q14_4 in [1,2,3,4,5,6]] Varlabel:In trees/	In trees/ woodlands/ grassy spaces (not including a garden at home or in other people's homes)

YouGov plc is a public limited company incorporated in England and Wales with company registration number 0367311 and with its registered office at 50 Featherstone Street, London, EC1Y 8RT. Website: www.yougov.com Tel: +44 207 012 6000 VAT Registration Number: 991 2281 09

woodlands/ grassy spaces (not including a garden at home or in other people's homes)	
-[GUC_Q15_5 if GUC_Q14_5 in [1,2,3,4,5,6]] Varlabel:In the street or public space close to your home (e.g. in your street, on the pavement in front of your home etc.) (not including local playgrounds)	In the street or public space close to your home (e.g. in your street, on the pavement in front of your home etc.) (not including local playgrounds)
-[GUC_Q15_6 if GUC_Q14_6 in [1,2,3,4,5,6]] Varlabel:Outdoors near water (e.g.at a beach, in the sea, near a river, lake or cliffs etc.)	Outdoors near water (e.g.at a beach, in the sea, near a river, lake or cliffs etc.)
-[GUC_Q15_7 if GUC_Q14_7 in [1,2,3,4,5,6]] Varlabel:At indoor play centres and pools (e.g. soft play, trampoline parks, swimming pools etc.)	At indoor play centres and pools (e.g. soft play, trampoline parks, swimming pools etc.)

<1>	Less than half an hour
<2>	Half an hour to 1 hour
<3>	1 to 2 hours
<4>	2 to 3 hours
<5>	3 to 4 hours
<6>	More than 4 hours
<977 fixed xor>	Don’t know/ can't recall

*Question type: **Text***

The following questions, by **'adventurous play'** we mean play that is thrilling and exciting where your child might experience some fear and uncertainty. This includes playing up high, moving so fast they feel they might lose control, playing near open water or a cliff, play fighting, playing with tools that could be dangerous or playing somewhere they could get lost and are out of sight of adults. For example, jumping off something very high, climbing high, riding a bike very fast, exploring woodland out of sight, would all be examples of adventurous play.

We are going to ask you how adventurously your child aged $Omnibus_Parents_recoded plays in each of the places you have told us that they play, using a scale outlined below:

• <u>Very low levels of adventure</u> = Play might be fun but levels of excitement, challenge and risk are low

• <u>Mild levels of adventure</u> = Some excitement but rarely feels any fear/ thrill, or takes any significant challenge and risk

YouGov plc is a public limited company incorporated in England and Wales with company registration number 0367311 and with its registered office at 50 Featherstone Street, London, EC1Y 8RT. Website: www.yougov.com Tel: +44 207 012 6000 VAT Registration Number: 991 2281 09

• Moderate levels of adventure = Excitement with some fear/ thrill and some minor challenge and risk taking

• High levels of adventure = Excitement with clear fear/ thrill and some minor challenge and risk taking

• Maximum levels of adventure = Very exciting with lots of thrilling emotions and fear and obvious challenge and risk

The button to move forward will appear after 15 seconds. Once it appears, please click this to continue.

Base: All GB parents of children aged 2-4
*Question type: **Grid***
#max number of choices per page: 4
[Varlabel - Adventurous play rating]

[GUC_Q16] You previously told us that your child plays in each of the following places...

Generally, how adventurously, if at all, does your child play in each of the following places? (Please select one option on each row)

-[GUC_Q16_1 if GUC_Q14_1 in [1,2,3,4,5,6] or GUC_Q12_1 in [1,2,3,4,5,6]] Varlabel:At home or in other people's homes	At home or in other people's homes
-[GUC_Q16_2 if GUC_Q14_2 in [1,2,3,4,5,6] or GUC_Q12_2 in [1,2,3,4,5,6]] Varlabel:Outside at home or at other people's homes (e.g. in a garden, yard, a balcony etc.)	Outside at home or at other people's homes (e.g. in a garden, yard, a balcony etc.)
-[GUC_Q16_3 if GUC_Q14_3 in [1,2,3,4,5,6] or GUC_Q12_3 in [1,2,3,4,5,6]] Varlabel:At a playground	At a playground
-[GUC_Q16_4 if GUC_Q14_4 in [1,2,3,4,5,6] or GUC_Q12_4 in [1,2,3,4,5,6]] Varlabel:In trees/ woodlands/ grassy spaces (not including a garden at home or in other people's homes)	In trees/ woodlands/ grassy spaces (not including a garden at home or in other people's homes)
-[GUC_Q16_5 if GUC_Q14_5 in [1,2,3,4,5,6] or GUC_Q12_5 in [1,2,3,4,5,6]] Varlabel:In the street or public space close to your home (e.g. in your street, on the pavement in front of your home etc.) (not including local playgrounds)	In the street or public space close to your home (e.g. in your street, on the pavement in front of your home etc.) (not including local playgrounds)

YouGov plc is a public limited company incorporated in England and Wales with company registration number 0367311 and with its registered office at 50 Featherstone Street, London, EC1Y 8RT. Website: www.yougov.com Tel: +44 207 012 6000 VAT Registration Number: 991 2281 09

-[GUC_Q16_6 if GUC_Q14_6 in [1,2,3,4,5,6] or GUC_Q12_6 in [1,2,3,4,5,6]] Varlabel:Outdoors near water (e.g.at a beach, in the sea, near a river, lake or cliffs etc.)	Outdoors near water (e.g.at a beach, in the sea, near a river, lake or cliffs etc.)
-[GUC_Q16_7 if GUC_Q14_7 in [1,2,3,4,5,6] or GUC_Q12_7 in [1,2,3,4,5,6]] Varlabel:At indoor play centres and pools (e.g. soft play, trampoline parks, swimming pools etc.)	At indoor play centres and pools (e.g. soft play, trampoline parks, swimming pools etc.)

<1>	Very low levels of adventure
<2>	Mild levels of adventure
<3>	Moderate levels of adventure
<5>	High levels of adventure
<6>	Maximum levels of adventure
<977 fixed xor>	Don’t know

Base: All GB parents of children aged 2-4
*Question type: **Single***
[Varlabel - Other place child plays adventurously]

[GUC_Q17] Is there another place not previously listed where your child plays adventurously? (Please select the option that best applies. If there is somewhere not previously listed where your child plays adventurously, please type it in the 'Yes, please specify' option. If there is more than one place not previously listed where your child plays adventurously, please think about the place they play the most)

<1>	Yes, there is (open [GUC_Q17_open1]) [open] please specify
<2>	No, there isn't
<977 fixed xor>	Don’t know

Base: All GB parents of children aged 2-4 whose child plays in an unlisted place
*Question type: **Grid***
#Question display logic:
If [GUC_Q17] - Yes, there is is selected [if GUC_Q17 == 1]
[Varlabel - Frequency of playing in seasons in unlisted place]

[GUC_Q18] You mentioned that there is somewhere not previously listed where your child plays adventurously...

How frequently, if at all, does your child play in this place, during each of the following times of year?

-[GUC_Q18_1] Varlabel:Autumn and Winter	Autumn and Winter (i.e. September to February)

YouGov plc is a public limited company incorporated in England and Wales with company registration number 0367311 and with its registered office at 50 Featherstone Street, London, EC1Y 8RT. Website: www.yougov.com Tel: +44 207 012 6000 VAT Registration Number: 991 2281 09

-[GUC_Q18_2] Varlabel:Spring and Summer	Spring and Summer (i.e. March to August)
<1>	Everyday
<2>	4 to 6 times a week
<3>	2 to 3 times a week
<4>	Once a week
<5>	Once a month
<6>	Less than once a month
<7>	Never
<977 fixed xor>	Don't know/ can't recall

Base: All GB parents of children aged 2-4 whose child plays in an unlisted place
*Question type: **Grid***
#Question display logic:
If [GUC_Q17] - Yes, there is is selected [if GUC_Q17 == 1]
[Varlabel - Time spent on a day playing in unlisted place]

[GUC_Q19] Thinking about a typical day that your child plays in this place, for approximately how long does you child play in each place in Spring and Summer? (Please select one option on each row)

-[GUC_Q19_1 if GUC_Q18_1 in [1,2,3,4,5,6]] Varlabel:Autumn and Winter	Autumn and Winter (i.e. September to February)
-[GUC_Q19_2 if GUC_Q18_2 in [1,2,3,4,5,6]] Varlabel:Spring and Summer	Spring and Summer (i.e. March to August)
<1>	Less than half an hour
<2>	Half an hour to 1 hour
<3>	1 to 2 hours
<4>	2 to 3 hours
<5>	3 to 4 hours
<6>	More than 4 hours
<977 fixed xor>	Don't know/ can't recall

Base: All GB parents of children aged 2-4 whose child plays in an unlisted place
*Question type: **Single***
#Question display logic:
If [GUC_Q17] - Yes, there is is selected [if GUC_Q17 == 1]
[Varlabel - Adventurous rating for unlisted play place]

[GUC_Q20] Still thinking about the place not previously listed where your child plays adventurously...

Generally, how adventurously, if at all, does your child play in this place? (Please select the option that best applies)

<1>	Very low levels of adventure

YouGov plc is a public limited company incorporated in England and Wales with company registration number 0367311 and with its registered office at 50 Featherstone Street, London, EC1Y 8RT. Website: www.yougov.com Tel: +44 207 012 6000 VAT Registration Number: 991 2281 09

<2>	Mild levels of adventure
<3>	Moderate levels of adventure
<5>	High levels of adventure
<6>	Maximum levels of adventure
<977 fixed xor>	Don't know

Base: All GB parents of children aged 2-4
*Question type: **Open***
#integer Only
[Varlabel - Age parent allowed out alone]

[GUC_Q27] Which factors, if any, allow you to let/ encourage your child to play in an adventurous way? (Please type your answer(s) in the box below, providing as much detail as possible)

Base: All GB parents of children aged 2-4
*Question type: **Open***
#any
[Varlabel - Factors that make it difficult to let children]

[GUC_Q27b] Which factors, if any, make it difficult for you to let/ encourage your child to spend more time playing in an adventurous way? (Please type your answer(s) in the box below, providing as much detail as possible)

Base: All GB parents of children aged 2-4
*Question type: **Grid***
#row order: reverse
[Varlabel - Time spent on a screen]

[GUC_Q30] For the following question, by an 'average weekday' we mean Monday to Friday, and by an 'average weekend day' we mean Saturday or Sunday.

Generally, how much time does your child spend looking at a screen (e.g. TV screen, tablet, phone etc.) throughout the week for each of the following purposes? (Please select one option on each row)

-[GUC_Q30_1] Varlabel:On an average weekday (Monday-Friday) for recreational purposes	On an average **weekday** for recreational/communication purposes
-[GUC_Q30_2] Varlabel:On an average weekend (Saturday-Sunday) for recreational purposes	On an average **weekend day** for recreational/communication purposes
-[GUC_Q30_3] Varlabel:On an average	On an average **weekday** for educational purposes

YouGov plc is a public limited company incorporated in England and Wales with company registration number 0367311 and with its registered office at 50 Featherstone Street, London, EC1Y 8RT. Website: www.yougov.com Tel: +44 207 012 6000 VAT Registration Number: 991 2281 09

weekday (Monday-Friday) for homework/ work purposes	
-[GUC_Q30_4] Varlabel:On an average weekend (Saturday-Sunday for homework/ work purposes	On an average **weekend day** for educational purposes
<1>	No time (i.e. 0 minutes)
<2>	Less than 1 hour
<3>	Between 1 to 2 hours
<4>	Between 2 to 3 hours
<5>	Between 3 to 4 hours
<6>	More than 4 hours
<977 fixed xor>	Don't know

*Question type: **Text***

The following questions will ask you about various topics related to your child, including your opinions towards what they do, what you allow them to do, and their emotions. They are all standardised measures within psychology that are used to asses behaviour; please do not worry if some of the questions seem daft or unrealistic, and try to give the first answer that comes to your head.

As a reminder, continue to think about your child aged $Omnibus_Parent_recoded.

Please click forward to continue.

Base: All GB parents of children aged 2-4
*Question type: **Grid***
#max number of choices per page: 5
[Varlabel - Child play risk agreement statements]

[GUC_Q28] Please indicate your level of agreement with the following statements.

-[GUC_Q28_1] Varlabel:I am concerned about the things I cannot control that can physically injure my child.	I am concerned about the things I cannot control that can physically injure my child.	-[GUC_Q28_8] Varlabel:I like to let my child find his or her own physical limits.	I like to let my child find his or her own physical limits.
-[GUC_Q28_2] Varlabel:Fewer injuries happen to children when parents plan	Fewer injuries happen to children when parents plan ways to prevent them.	-[GUC_Q28_9] Varlabel:I value opportunities for my child to explore new environments.	I value opportunities for my child to explore new environments.

YouGov plc is a public limited company incorporated in England and Wales with company registration number 0367311 and with its registered office at 50 Featherstone Street, London, EC1Y 8RT. Website: www.yougov.com Tel: +44 207 012 6000 VAT Registration Number: 991 2281 09

ways to prevent them.			
-[GUC_Q28_3] Varlabel:I am concerned about the potential hazards in my home.	I am concerned about the potential hazards in my home.	-[GUC_Q28_10] Varlabel:Benefits of physical activity for my child outweigh the risk of experiencing minor injuries.	Benefits of physical activity for my child outweigh the risk of experiencing minor injuries.
-[GUC_Q28_4] Varlabel:Children should play in places where there is low risk of injury.	Children should play in places where there is low risk of injury.	-[GUC_Q28_11] Varlabel:I prefer to teach my child how to manage risky situations rather than avoid them.	I prefer to teach my child how to manage risky situations rather than avoid them.
-[GUC_Q28_5] Varlabel:Good supervision of my child means knowing where my child is going at all times.	Good supervision of my child means knowing where my child is going at all times.	-[GUC_Q28_12] Varlabel:Participating in challenging and potentially risky physical activities will help my child develop self-confidence.	Participating in challenging and potentially risky physical activities will help my child develop self-confidence.
-[GUC_Q28_6] Varlabel:Letting my child engage in physical activities without supervision greatly increases their chance of injury.	Letting my child engage in physical activities without supervision greatly increases their chance of injury.	-[GUC_Q28_13] Varlabel:It feels as if I'm always driving my child to an organised sport or activity.	It feels as if I'm always driving my child to an organised sport or activity.
-[GUC_Q28_7] Varlabel:It is important for my child to engage in physically challenging experiences.	It is important for my child to engage in physically challenging experiences.	-[GUC_Q28_14] Varlabel:The number of organised sports or activities my child participates in is a source of stress for the family.	The number of organised sports or activities my child participates in is a source of stress for the family.

<1>	Very strongly disagree
<2>	Strongly disagree
<3>	Disagree
<4>	Neither agree nor disagree
<5>	Agree
<6>	Strongly agree
<7>	Very strongly agree

YouGov plc is a public limited company incorporated in England and Wales with company registration number 0367311 and with its registered office at 50 Featherstone Street, London, EC1Y 8RT. Website: www.yougov.com Tel: +44 207 012 6000 VAT Registration Number: 991 2281 09

Base: All GB parents of children aged 2-4
*Question type: **Grid***
#max number of choices per page: 7
[Varlabel - Allowing children to do each factor]

[GUC_Q29] Please answer yes or no to the following questions.

-[GUC_Q29_1] Varlabel:Would you let your child jump down from a height of 3-4m	Would you let your child jump down from a height of 3-4m	-[GUC_Q29_17] Varlabel:Would you let your child run close to an open fire	Would you let your child run close to an open fire
-[GUC_Q29_2] Varlabel:Would you allow your child to play chase with other children	Would you allow your child to play chase with other children	-[GUC_Q29_18] Varlabel:Would you let your child swim in the sea close to the shore while you were watching from the beach	Would you let your child swim in the sea close to the shore while you were watching from the beach
-[GUC_Q29_3] Varlabel:Would you trust your child to play by themselves without constant supervision	Would you trust your child to play by themselves without constant supervision	-[GUC_Q29_19] Varlabel:Would you allow your child to continue playing if there is potential they may break a bone	Would you allow your child to continue playing if there is potential they may break a bone
-[GUC_Q29_4] Varlabel:Would you let your child go head first down a slide	Would you let your child go head first down a slide	-[GUC_Q29_20] Varlabel:Would you let your child play in a backyard unsupervised	Would you let your child play in a backyard unsupervised
-[GUC_Q29_5] Varlabel:Would you allow your child to continue playing if they get a few	Would you allow your child to continue playing if they get a few scrapes during play	-[GUC_Q29_21] Varlabel:Would you allow your child to play-fight, testing who is strongest	Would you allow your child to play-fight, testing who is strongest

YouGov plc is a public limited company incorporated in England and Wales with company registration number 0367311 and with its registered office at 50 Featherstone Street, London, EC1Y 8RT. Website: www.yougov.com Tel: +44 207 012 6000 VAT Registration Number: 991 2281 09

scrapes during play			
-[GUC_Q29_6] Varlabel:Would you let your child have lots of challenges when they play at home	Would you let your child have lots of challenges when they play at home	-[GUC_Q29_22] Varlabel:Would you allow your child to climb a rock wall that goes straight down to the water	Would you allow your child to climb a rock wall that goes straight down to the water
-[GUC_Q29_7] Varlabel:Would you let your child use a hammer and nail unsupervised	Would you let your child use a hammer and nail unsupervised	-[GUC_Q29_23] Varlabel:Would you wait to see if your child could manage challenges on their own before getting involved	Would you wait to see if your child could manage challenges on their own before getting involved
-[GUC_Q29_8] Varlabel:Would you let your child climb up a tree in your reach	Would you let your child climb up a tree in your reach	-[GUC_Q29_24] Varlabel:Would you let your child climb as high as they want to in trees	Would you let your child climb as high as they want to in trees
-[GUC_Q29_9] Varlabel:Would you let your child walk barefoot across a floor after broken glass had been swept up	Would you let your child walk barefoot across a floor after broken glass had been swept up	-[GUC_Q29_25] Varlabel:Would you allow your child to ride a bicycle down a steep hill at full speed	Would you allow your child to ride a bicycle down a steep hill at full speed
-[GUC_Q29_10] Varlabel:Would you let your child walk on slippery rocks close to water	Would you let your child walk on slippery rocks close to water	-[GUC_Q29_26] Varlabel:Would you trust your child to play safely	Would you trust your child to play safely
-[GUC_Q29_11]	Would you allow your child to play-fight other children with sticks	-[GUC_Q29_27]	Would you allow your child to use a sharp knife

YouGov plc is a public limited company incorporated in England and Wales with company registration number 0367311 and with its registered office at 50 Featherstone Street, London, EC1Y 8RT. Website: www.yougov.com Tel: +44 207 012 6000 VAT Registration Number: 991 2281 09

Varlabel:Would you allow your child to play-fight other children with sticks		Varlabel:Would you allow your child to use a sharp knife	
- [GUC_Q29_12] Varlabel:Would you encourage your child to try new things that involve some risk	Would you encourage your child to try new things that involve some risk	- [GUC_Q29_28] Varlabel:Would you let your child play in a garden unsupervised	Would you let your child play in a garden unsupervised
- [GUC_Q29_13] Varlabel:Would you allow your child to engage in rough and tumble play	Would you allow your child to engage in rough and tumble play	- [GUC_Q29_29] Varlabel:Would you let your child balance on a fallen tree more than 2m above the ground	Would you let your child balance on a fallen tree more than 2m above the ground
- [GUC_Q29_14] Varlabel:Would you let your child play near the edge of steep cliffs	Would you let your child play near the edge of steep cliffs	- [GUC_Q29_30] Varlabel:Would you encourage your child to take some risks if it means having fun during play	Would you encourage your child to take some risks if it means having fun during play
- [GUC_Q29_15] Varlabel:Would you allow your child to play in the woods out of your sight	Would you allow your child to play in the woods out of your sight	- [GUC_Q29_31] Varlabel:Would you allow your child to climb up a tree beyond your reach	Would you allow your child to climb up a tree beyond your reach
- [GUC_Q29_16] Varlabel:Would you let your child experience minor mishaps	Would you let your child experience minor mishaps if what they are doing is lots of fun		

YouGov plc is a public limited company incorporated in England and Wales with company registration number 0367311 and with its registered office at 50 Featherstone Street, London, EC1Y 8RT. Website: www.yougov.com Tel: +44 207 012 6000 VAT Registration Number: 991 2281 09

if what they are doing is lots of fun	
<1>	Yes
<2>	No
<977 fixed xor>	Don't know

*Question type: **Text***

For the following question, please think about your child aged $Omnibus_Parents_recoded. Please think about their behaviour over the last 6 months.

Please click forward to continue.

Has your child ever suffered an injury whilst playing outside that required treatment from a healthcare professional at hospital? This might include attending A&E for treatment for a fracture/ sprain, head injury that required observation and/or scan, or cuts that required wound closure with glue, steri-strips or stitches etc.

<1>	Yes (if so, what)
<2>	No
<977 fixed xor>	Prefer not to say

Base: All GB parents of children aged 2-4
*Question type: **Grid***
[Varlabel - SQD measure]

[GUC_Q33] Strengths and Difficulties Questionnaire

For each item, please tick the box for Not True, Somewhat True or Certainly True. It would help us if you answered all items as best you can even if you are not absolutely certain or the item seems daft! Please give your answers on the basis of the child's behaviour over the last 6 months or this school year.

-[GUC_Q33_1] Varlabel:Considerate of other people's feeling	Considerate of other people's feeling	-[GUC_Q33_14] Varlabel:Generally liked by other children	Generally liked by other children
-[GUC_Q33_2] Varlabel:Restless, overactive, cannot stay still for long	Restless, overactive, cannot stay still for long	-[GUC_Q33_15] Varlabel:Easily distracted, concentration wanders	Easily distracted, concentration wanders

YouGov plc is a public limited company incorporated in England and Wales with company registration number 0367311 and with its registered office at 50 Featherstone Street, London, EC1Y 8RT. Website: www.yougov.com Tel: +44 207 012 6000 VAT Registration Number: 991 2281 09

-[GUC_Q33_3] Varlabel:Often complains of headaches, stomach-aches or sickness	Often complains of headaches, stomach-aches or sickness	-[GUC_Q33_16] Varlabel:Nervous or clingy in new situations, easily loses confidence	Nervous or clingy in new situations, easily loses confidence
-[GUC_Q33_4] Varlabel:Shares readily with other children (treats, toys, pencils etc.)	Shares readily with other children (treats, toys, pencils etc.)	-[GUC_Q33_17] Varlabel:Kind to younger children	Kind to younger children
-[GUC_Q33_5] Varlabel:Often has temper tantrums or hot tempers	Often has temper tantrums or hot tempers	-[GUC_Q33_18] Varlabel:Often lies or cheats	Often argumentative with adults
-[GUC_Q33_6] Varlabel:Rather solitary, tends to play alone	Rather solitary, tends to play alone	-[GUC_Q33_19] Varlabel:Picked on or bullied by other children	Picked on or bullied by other children
-[GUC_Q33_7] Varlabel:Generally obedient, usually does what adults request	Generally obedient, usually does what adults request	-[GUC_Q33_20] Varlabel:Often volunteers to help others (parents, teachers, other children)	Often volunteers to help others (parents, teachers, other children)
-[GUC_Q33_8] Varlabel:Many worries, often seems worried	Many worries, often seems worried	-[GUC_Q33_21] Varlabel:Thinks things out before acting	Can stop and think things out before acting
-[GUC_Q33_9] Varlabel:Helpful if someone is hurt, upset or feeling ill	Helpful if someone is hurt, upset or feeling ill	-[GUC_Q33_22] Varlabel:Steals from home, school or elsewhere	Can be spiteful to others
-[GUC_Q33_10] Varlabel:Constantly fidgeting or squirming	Constantly fidgeting or squirming	-[GUC_Q33_23] Varlabel:Gets on better with adults than with other children	Gets on better with adults than with other children
-[GUC_Q33_11] Varlabel:Has at least one good friend	Has at least one good friend	-[GUC_Q33_24] Varlabel:Many fears, easily scared	Many fears, easily scared
-[GUC_Q33_12] Varlabel:Often fights with other children or bullies them	Often fights with other children or bullies them	-[GUC_Q33_25] Varlabel:Sees tasks through to	Sees tasks through to the end, good attention span

YouGov plc is a public limited company incorporated in England and Wales with company registration number 0367311 and with its registered office at 50 Featherstone Street, London, EC1Y 8RT. Website: www.yougov.com Tel: +44 207 012 6000 VAT Registration Number: 991 2281 09

		the end, good attention span
-[GUC_Q33_13] Varlabel:Often unhappy, down-hearted or tearful	Often unhappy, down-hearted or tearful	

<1> Not True
<2> Somewhat True
<3> Certainly True

*Question type: **Text***

Thank you very much for your help

[IMAGE /copylogo.JPG]

*Question type: **Text***

For the next question, we would like you to think about the past few weeks

Base: All GB parents of children aged 2-4
*Question type: **Grid***
#max number of choices per page: 5
[Varlabel - Feelings of child over past few weeks]

[GUC_Q32] This scale has a number of words that describe different feelings and emotions.

Read each item and then select the best answer next to that word. Indicate to what extent your child has felt this way **during the past few weeks**. There are no right or wrong answers.

-[GUC_Q32_1] Varlabel:Sad	Sad
-[GUC_Q32_2] Varlabel:Happy	Happy
-[GUC_Q32_3] Varlabel:Scared	Scared
-[GUC_Q32_4] Varlabel:Miserable	Miserable
-[GUC_Q32_5] Varlabel:Cheerful	Cheerful
-[GUC_Q32_6] Varlabel:Proud	Proud
-[GUC_Q32_7] Varlabel:Afraid	Afraid
-[GUC_Q32_8] Varlabel:Joyful	Joyful

YouGov plc is a public limited company incorporated in England and Wales with company registration number 0367311 and with its registered office at 50 Featherstone Street, London, EC1Y 8RT. Website: www.yougov.com Tel: +44 207 012 6000 VAT Registration Number: 991 2281 09

-[GUC_Q32_9] Mad
Varlabel:Lonely
-[GUC_Q32_10] Lively
Varlabel:Lively

<1>	Very slightly or not at all
<2>	A little
<3>	Moderately
<4>	Quite a bit
<5>	Extremely

Base: All GB parents of children aged 2-4
*Question type: **Single***
[Varlabel - Personal feeling warning]

[GUC_Q31_opener] On the next page we would like to ask you how you personally have been feeling over the past year.

Specifically, this question will ask you how often you have felt six negative emotions. It is a standardised mental health measure in psychology, and for this reason you will not be provided with a 'Prefer not to say' option.
If you would prefer to not complete this section, we have provided the option to skip this question below, and if you do choose to proceed, you will have the option to not provide answers on any of the rows if you would rather not give an answer.
Are you happy to proceed with answering this question?

<1>	Yes, I am
<2>	No, I am not

Base: All GB parents of children aged 2-4 happy to complete question
*Question type: **Grid***
#column order: reverse
#Question display logic:
If [GUC_Q31_opener] - Yes, I am is selected [if GUC_Q31_opener == 1]
[Varlabel - Feelings personally over the past year]

[GUC_Q31] The following questions ask about how you have been feeling during **past year.**

For each question, please select the number that best describes how often you had this feeling.

During the past year, about how often did you feel...
-[GUC_Q31_1] Nervous
Varlabel:Nervous
-[GUC_Q31_2] Hopeless
Varlabel:Hopeless

YouGov plc is a public limited company incorporated in England and Wales with company registration number 0367311 and with its registered office at 50 Featherstone Street, London, EC1Y 8RT. Website: www.yougov.com Tel: +44 207 012 6000 VAT Registration Number: 991 2281 09

-[GUC_Q31_3] Varlabel:Restless or fidgety	Restless or fidgety
-[GUC_Q31_4] Varlabel:So depressed that nothing could cheer you up	So depressed that nothing could cheer you up
-[GUC_Q31_5] Varlabel:That everything was an effort	That everything was an effort
-[GUC_Q31_6] Varlabel:Worthless	Worthless
<1>	(1) All of the time
<2>	(2) Most of the time
<3>	(3) Some of the time
<4>	(4) A little of the time
<5>	(5) None of the time

*Question type: **Text***

Thank you for taking part in this survey. If you've been affected by this topic and would like any more information, need advice, or support, you can go to any of the following places for help:

Samaritans - www.samaritans.org - call 116 123 (24 hour helpline)

Mind – www.mind.org.uk - call 0300 123 3393 (Monday to Friday 9am - 6pm, except for bank holidays)

Papyrus - www.papyrus-uk.org – call 0800 068 4141 (Monday to Friday 10am-10pm, weekends: 2pm-10pm and bank holidays 2pm-5pm)

PROJECT DETAILS

TIMINGS

Project sign off required by 3pm:	
Project set up:	
Fieldwork commences:	
Expected delivery:	
Profiles delivery:	

SAMPLE

YouGov plc is a public limited company incorporated in England and Wales with company registration number 0367311 and with its registered office at 50 Featherstone Street, London, EC1Y 8RT. Website: www.yougov.com Tel: +44 207 012 6000 VAT Registration Number: 991 2281 09

**Size:**	**c.1000 parents of children aged 2-4**
**Description:**	**Fallout from a Nat-rep sample**

YouGov plc is a public limited company incorporated in England and Wales with company registration number 0367311 and with its registered office at 50 Featherstone Street, London, EC1Y 8RT. Website: www.yougov.com Tel: +44 207 012 6000 VAT Registration Number: 991 2281 09
